# Periodontal Risk, Self-Reported Quality of Life, and Sports Performance: A Cross-Sectional Study of Japanese University Athletes

**DOI:** 10.3390/sports14010018

**Published:** 2026-01-04

**Authors:** Rena Hamano, Manabu Nakata, Makoto Nakadake, Akira Nakamura, Yoshimitsu Kohmura, Kazuhiro Aoki, Keisuke Sawaki, Hidefumi Waki, Tomonori Kito, Yoshio Suzuki

**Affiliations:** 1 Faculty of Health and Sports Science, Juntendo University, Inzai 270-1695, Japan; r.hamano.uo@juntendo.ac.jp (R.H.); m-nakata@juntendo.ac.jp (M.N.); mknakada@juntendo.ac.jp (M.N.); aknaka@juntendo.ac.jp (A.N.); ykoumura@juntendo.ac.jp (Y.K.); k-aoki@juntendo.ac.jp (K.A.); ksawaki@juntendo.ac.jp (K.S.); hwaki@juntendo.ac.jp (H.W.); t.kito@juntendo.ac.jp (T.K.); 2 Graduate School of Health and Sports Science, Juntendo University, Inzai 270-1695, Japan

**Keywords:** periodontal disease, athlete health, oral health impact, TLP-AA kit, red complex bacteria, Oral Impacts on Daily Performance (OIDP), Immune Status Questionnaire (ISQ)

## Abstract

Background: Periodontal disease is prevalent among elite athletes globally, yet its status in Japanese athletes remains unclear. This study assessed periodontal risk in Japanese university athletes and its association with oral health-related quality of life (QOL), sports performance, and immune status. Methods: A cross-sectional study was conducted with 313 university athletes (basketball, volleyball, athletics). Periodontal risk was evaluated using a Trypsin-Like Peptidase activity assay (TLP-AA) kit; a score ≥ 1.5 indicated the presence of Red Complex bacteria (positive risk). Participants completed the Oral Impacts on Daily Performance (OIDP), Oslo Sports Trauma Research Centre (OSTRC) overuse injury, and Immune Status Questionnaires (ISQ). Results: Positive periodontal risk was identified in 25.9% (81/313) of athletes. While overall prevalence was not statistically different from the general population (17.8%), specific associations emerged. Positive risk was significantly associated with “pain related to sports” (OSTRC) overall (*p* = 0.016) and specifically in males (*p* = 0.004). Among track and field athletes, positive risk was associated with difficulty “smiling/laughing” (OIDP, *p* = 0.033) and lower self-rated general health (*p* = 0.032) and immune functioning (*p* = 0.047). Conclusions: Periodontal risk is notable in Japanese university athletes and is significantly associated with sports-related pain and specific QOL domains. Regular oral health monitoring may be crucial for maintaining athletic performance and well-being.

## 1. Introduction

A considerable proportion of the athletes who participated in the 2012 London Olympics—of whom the majority were from Africa, the Americas, and Europe—reported marked deterioration in oral health. This included dental caries (55%), dental erosion (45%), and periodontal disease (gingivitis in 76% and periodontitis in 15% of cases) [1]. A later study of professional/elite football players in the United Kingdom (UK) found that 37% had active caries, 53% dental erosion, and 5% moderate-to-severe irreversible periodontal disease [2]. Furthermore, a 2018 study examining the association between periodontal health and self-reported oral health problems among elite athletes (352 individuals across 11 sports) in the UK found a high prevalence of oral health issues, with 49.1% experiencing caries and 77.0% bleeding gums or tartar [3]. Additionally, 32.0% of athletes reported that their oral health impacted their sports performance [3]. Furthermore, athletes exhibiting signs or symptoms of periodontal disease are more likely to experience recurrent injuries [4]. However, the number of such studies remains limited, and none have been conducted on Japanese athletes.

A diagnostic kit for measuring trypsin-like peptidase activity in morning tongue-swab fluid has recently been developed, and it has been reported to be able to assess the risk of periodontal disease [5,6,7]. The trypsin-like peptidase activity assay kit (TLP-AA kit) measures the trypsin-like protease levels produced by the three bacteria of the red complex (*Porphyromonas gingivalis*, *Tannerella forsythia*, and *Treponema denticola*) causing periodontitis [8]. This enables the accurate detection of these bacteria in samples.

This study used the TLP-AA kit to investigate the risk of periodontal disease among Japanese university athletes. We also examined the association between self-reported oral health issues and performance. that periodontal disease may be of a higher prevalence among Japanese university athletes than among their Japanese peers and might have a negative impact on their self-reported quality of life and athletic performance.

## 2. Materials and Methods

### 2.1. Study Setting and Participants

This cross-sectional study targeted athletes at Juntendo University in the basketball, volleyball, and athletics clubs. The study outline, including its purpose, methods, and expected outcomes, was communicated to them via a webpage distributed by club coaches. Participants were asked to read the research content on the study’s website and provide their consent by checking the consent box on the linked response page (created in Google Docs), after which they proceeded to answer the questionnaires. They were also provided with a tongue-swab sampling kit, the manufacturer’s instructions, and a link to a video explaining the collection method via their club coaches. They collected their own tongue swabs upon waking, placed them in the extraction buffer, and stirred, following the protocol described. The club leaders delivered the samples to the researchers within 4 h of collection at an ambient temperature of less than 20 °C and were stored at 4 °C. The samples were delivered to the manufacturer in a cooled state within 30 days of collection for TLP-AA evaluation. The study was conducted from November to December 2024.

This study did not involve obtaining new samples or information through invasive procedures or interventions. This study was conducted under the informed consent procedures specified in the Japanese government’s Ethical Guidelines for Life Science and Medical Research Involving Human Participants (last revised on 27 March 2023), although written informed consent was not required in this case. However, informed consent had to be obtained regarding the items specified in the guidelines. Records detailing the method and content of the explanation provided, as well as the consent obtained, had to be kept. The study was approved by the Juntendo University Ethics Committee (Approval No. 2024-69) and was conducted in accordance with the guidelines of the Declaration of Helsinki.

### 2.2. Questionnaires

Participants answered questions about their attributes (name, age, gender, height, weight, and affiliated club) and their general quality of life (QOL), oral health–related impact on sporting performance, and immune status on the response page.

Questions regarding general QOL were based on the Oral Impacts on Daily Performance (OIDP) outcome measure used in the Adult Dental Health Survey (ADHS 2009) in England, Wales, and Northern Ireland [9]. Three items (difficulty eating or drinking, relaxing including sleeping, and laughing or showing teeth without embarrassment) were selected as highly relevant to the young, healthy population. This set has also been used by Gallagher et al. [3] to investigate the association between oral health and performance in elite athletes in the UK.

The impact of oral health on sporting performance was assessed using the Oslo Sports Trauma Research Centre (OSTRC) overuse injury questionnaire [4]; Gallagher et al. [3].

The immune status was assessed using the seven-item Immune Status Questionnaire (ISQ), which was developed to score immune status, and an 11-point numerical rating scale (NRS) for general health and immune functioning [10].

### 2.3. Statistical Analysis

The 95% confidence interval (95% CI) for the risk of periodontal disease was calculated using the Clopper–Pearson exact method, based on the binomial distribution. The Shapiro–Wilk test was used to confirm normality.

Because the response distributions were skewed, the questions with the majority of responses (≥70%) of OIDP and the OSTRC were compared with the others. For the NRS of the ISQ, the responses were dichotomized based on the median and evaluated for their association with periodontal risk. Where significant differences were observed, the original NRS scores were compared using the Mann–Whitney U test.

A series of 2 × 2 contingency tables was analyzed using Fisher’s Exact Test, an independent single-hypothesis test. Differences in periodontal risk between clubs were analyzed using a 2 × 3 contingency table and the chi-squared test. However, as the overall result was not significant, no post hoc analysis was performed. Differences in ISQ scores between periodontal risk groups (negative and positive) were determined using the Mann–Whitney U test.

In line with Gallagher et al. [3], comparisons were stratified by gender and club, but not by their interaction. This was because of the limited sample size available for statistical analysis.

Statistical analysis was performed using SPSS version 29 (IBM Japan, Tokyo, Japan). The significance level was set at 5%.

## 3. Results

### 3.1. Participants

Overall, 52.7% (313 out of 594) of subjects participated in the trial. The participation rates by club were as follows: 64.2% (95/148) for basketball; 94.9% (75/79) for volleyball; and 39.0% (143/367) for athletics. Table 1 summarizes the number of participants and their age, height, and weight.

### 3.2. Periodontal Risk

Table 2 presents the participants’ TLP-AA kit scores, as measured by the TLP-AA kits, stratified by gender and club. This diagnostic tool classifies TLP-AA into seven levels: 1, 1.5, 1.75, 2, 3, 4, and 5. However, the maximum score among the participants in this study was 2, with scores of 3 or higher not observed.

Over 70% of both female and male respondents scored 1 and did not have the red complex bacteria. Therefore, periodontal risk was classified into two categories: positive for those assessed as having the red complex (TLP-AA kit score ≥ 1.5) and negative for those assessed as not having it (TLP-AA kit score < 1.5).

**Figure 1:**
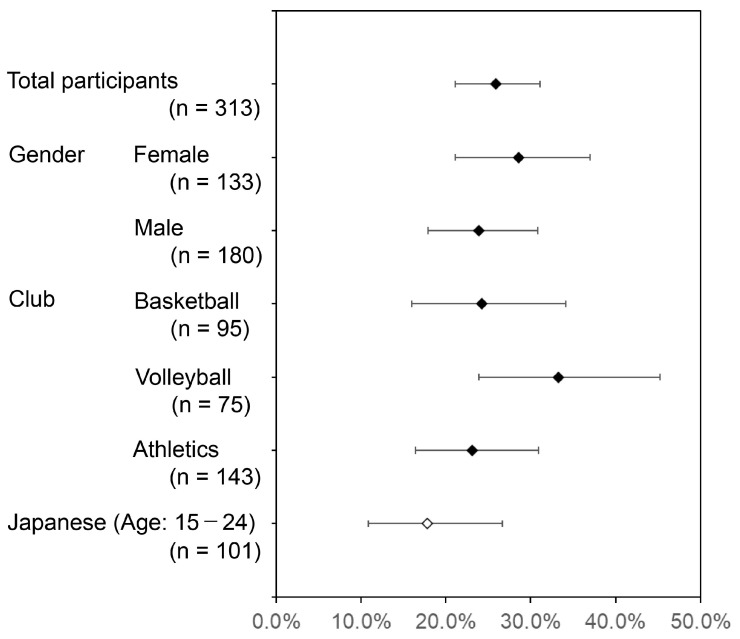
Comparison of periodontal risk prevalence between university athletes (TLP-AA kit score ≥ 1.5) and age-matched general Japanese population (PPD ≥ 4 mm). Black diamonds represent the prevalence of periodontal risk (positive) in the university athlete cohort (n = 313), defined by a TLP-AA kit score of ≥1.5. The error bars surrounding the black diamonds indicate the 95% Confidence Intervals (CI) for the athlete group. The white diamond represents the reported prevalence of periodontal pockets ≥ 4 mm in the age-matched general Japanese population, and the associated error bars indicate the 95% CI.

Figure 1 shows the percentage and 95% CI of participants with a positive result. There was no significant difference in periodontal risk by gender (*p* = 0.363). A chi-square test revealed no significant differences among the three affiliated clubs in the proportions of participants with a positive result (*p* = 0.235).

For reference, Figure 1 also shows the proportion of Japanese individuals aged 15–24 with periodontal pocket depth (PPD) of 4 mm or greater (17.8%; 95% CI 10.9–26.7%), as reported in the Ministry of Health, Labor, and Welfare’s 2022 Summary of Dental Disease Survey Results [11]. Although the proportion of positive cases in this study was higher than 17.8% overall, when stratified by gender and club, the 95% CIs indicated no significant differences.

### 3.3. Periodontal Risk and the General Quality of Life

Responses for general QOL were scored on a 6-point scale ranging from 0 (no problem) to 5 (very difficult) for the three items selected from the OIDP. Over 70% of the responses to all three items were scored 0, and the remaining responses were grouped into a single “difficulty” category, and the resulting binary values were analyzed.

No significant relationship was found between the responses to the questions and periodontal risk. However, approximately 10% more athletes with a positive periodontal risk reported difficulty in “smiling, laughing, or showing teeth without embarrassment” than did those with a negative risk, 34.6% and 25.9%, respectively (*p* = 0.152, Table 3).

**Figure 2:**
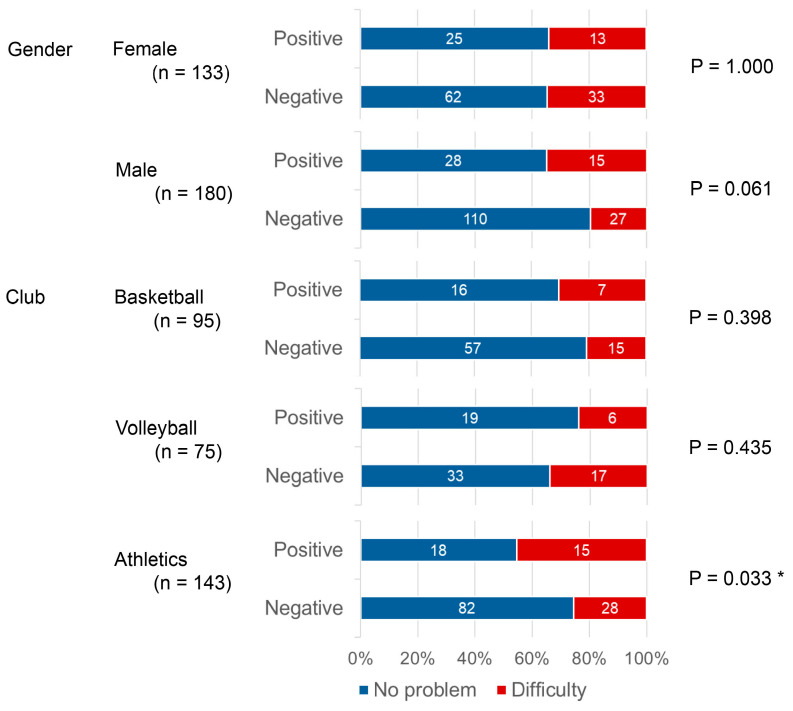
Association between periodontal risk status and Self-Reported Oral Impacts on Daily Performance (OIDP) scores: Difficulty in smiling, laughing, or showing teeth without embarrassment (OIDP Item 3). The bar chart illustrates the distribution of responses (“No problem” or “Difficulty”) to the OIDP item concerning smiling, laughing, or showing teeth without embarrassment, stratified by periodontal risk status (Positive: TLP-AA kit score ≥ 1.5; Negative: TLP-AA kit score < 1.5). The numbers within the bars indicate the number of athletes (*n*) whose responses were categorized as “No problem” or “Difficulty.” The association between periodontal risk and the responses was assessed using Fisher’s Exact Test. * An asterisk indicates a statistically significant difference in proportions between the positive and negative periodontal risk groups (*p* < 0.05).

When the responses to the question “Smiling, laughing, or showing teeth without embarrassment” were stratified by gender and club, the proportion of those reporting difficulty was markedly higher among those in athletics with a positive than a negative periodontal risk (*p* = 0.033, Figure 2).

No significant relationships were observed in the responses to the other two questions by gender or club.

### 3.4. Periodontal Risk and the Impact of Oral Health on Sporting Performance

Regarding the impact of oral health on sports performance, the OSTRC overuse injury questionnaire asked participants to reflect on their past week and rate the extent to which oral health issues affected their participation, training volume, performance, and sports-related pain on a 4- or 5-point scale. Since over 85% of the responses to all four questions indicated “no effect,” those responses were classified as “Unaffected,” and all other responses were grouped as “Affected” to create a binary classification.

Comparison of the responses with periodontal risk revealed that the proportion of “affected” responses for “pain related to sports” was significantly higher in the positive (18.5%) than in the negative group (8.6%) (*p* = 0.016, Table 4).

**Figure 3:**
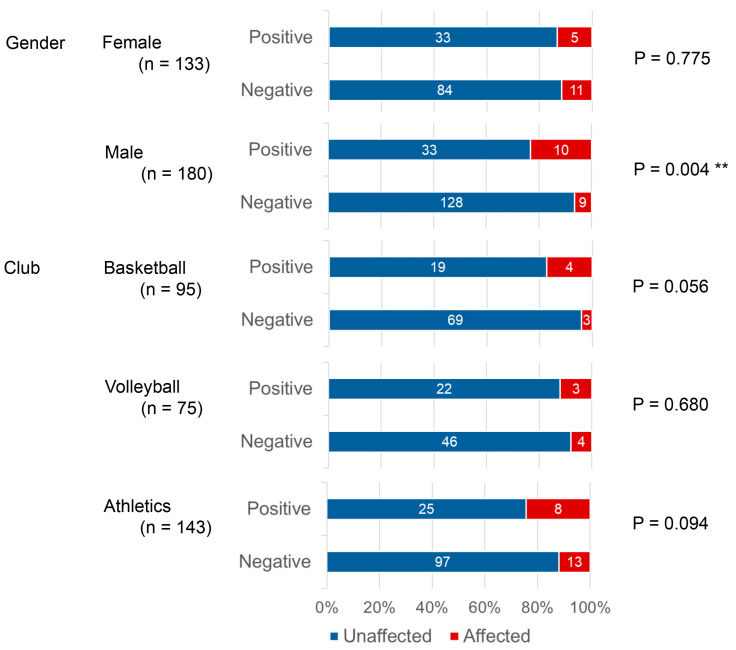
Comparison of self-rated general health status (dichotomized NRS) by periodontal risk status and club. The bar chart illustrates the distribution of the dichotomized self-rated general health status (NRS) responses, stratified by periodontal risk status (Positive: TLP-AA kit score ≥ 1.5; Negative: TLP-AA kit score < 1.5) and the athlete’s club (Track and Field, Basketball, Volleyball). The scores were dichotomized into “Poor” (NRS < 7) and “Good” (NRS ≥ 7). The numbers within the bars indicate the number of athletes (*n*) who responded in each category. The association between periodontal risk and the dichotomized NRS score was assessed using Fisher’s Exact Test for each club independently. ** Double asterisks indicate a statistically significant difference in proportions between the periodontal risk Positive and Negative groups within the specific club (*p* < 0.01).

When responses for sports-related pain were stratified by gender and club, male respondents had a significantly higher proportion of affected individuals compared to those with a negative risk (*p* = 0.004). For each of the three clubs individually, the proportion of affected individuals was higher among those with a positive than with a negative periodontal risk, but not statistically significantly so (Figure 3).

No significant relationships by gender or club were observed for the other three items.

### 3.5. Periodontal Risk and Immune Status

Respondents rated how often they experienced “sudden high fever,” “diarrhea,” “headache,” “skin problems (e.g., acne & eczema),” “muscle and joint pain,” “common cold,” and “coughing” in the past 12 months on a scale ranging from 0 (never experienced) to 4 (almost always experienced). The sum of the response scores constitutes the ISQ score [10].

The median ISQ score (25th percentile; 75th percentile) for all participants was 7.0 (4.0, 9.0). Using the Shapiro–Wilk test, normality could not be confirmed for either the Positive (*p* = 0.037) or Negative (*p* < 0.001) groups when classified by periodontal risk. The median (25th percentile; 75th percentile) was 6.0 (3.0, 8.5) for the positive group and 7.0 (4.0, 9.0) for the negative group. A Mann–Whitney U test showed no significant difference in the medians of the two groups (*p* = 0.547).

In the aforementioned paper [10], participants completed the ISQ, as well as two questions about (A) perceived immune functioning and (B) perceived general health on an 11-point scale ranging from 0 (very poor) to 10 (excellent). Therefore, this study included the same questions. The responses to neither question could be assumed to be normally distributed (both *p* < 0.001). Both had a median of 7.0, with the mean slightly skewed toward lower values: 6.1 for general health and 6.0 for immune functioning. Thus, responses below 7 points were coded as “poor and the others as “good,” thus using the median as the cutoff.

**Figure 4:**
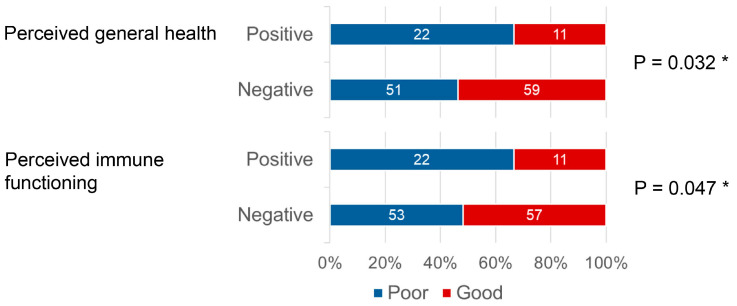
Comparison of self-rated general health and immune functioning (dichotomized NRS) by periodontal risk status and club. The bar chart illustrates the distribution of the self-rated general health immune functioning (NRS) responses, stratified by periodontal risk status (Positive: TLP-AA kit score ≥ 1.5; Negative: TLP-AA kit score < 1.5) and the athlete’s club (basketball, volleyball, and athletics), with scores dichotomized into “Poor” (NRS < 7) and “Good” (NRS ≥ 7). The numbers within the bars indicate the number of athletes (*n*) who responded in each category. The association between periodontal risk and the dichotomized NRS score was assessed using Fisher’s Exact Test for each club independently. * An asterisk indicates a statistically significant difference in proportions between the positive and negative periodontal risk groups within a particular club (*p* < 0.05).

No significant differences were found overall for the association of periodontal risk with general health (*p* = 0.266) or immune functioning (*p* = 0.157). However, when stratified by gender and club in athletics, the proportion of good scores was significantly lower than that of poor scores for both general health (*p* = 0.032) and immune functioning (*p* = 0.047) among those with positive periodontal disease (Figure 4). Mann–Whitney U test comparing the pre-binary scores for general health status and immune functioning in athletics revealed no significant difference for general health status (*p* = 0.088) but a significant difference for immune functioning (*p* = 0.047).

## 4. Discussion

This study measured the periodontal risk of Japanese University athletes using the TLP-AA kit and examined its association with their general quality of life (QOL), the impact of oral health on their sporting performance, and their immune status.

The TLP-AA kit measures the activity of TLPs produced by oral red complex species with 90% specificity and 92% accuracy [8], where a score of 1.5 or higher indicates a 97% probability of the presence of oral red complex species, with a positive agreement rate of 97% [8]. The TLP-AA kit score correlates with the degree of periodontal risk. Moderate periodontal disease, as defined by the CDC/AAP, is defined as “moderate periodontitis: At least two sites (not on the same tooth) with subgingival bone loss (CAL) of at least 4 mm in the interproximal region or at least two sites (not on the same tooth) with a PPD of at least 5 mm” (CDC/AAP). TLP-AA kit score values (median, IQR) for severe and moderate periodontitis were 2.25 (2.0–3.0) and 1.25 (1.0–2.25), respectively. These values are significantly higher than the score of 1.0 (1.0–1.5) for “no periodontal disease” [5]. However, according to the CDC/AAP definition, mild periodontal disease is characterized by subgingival bone loss (CAL) of 3 mm or more in at least two interproximal sites (not on the same tooth) and a PPD of 4 mm or more in at least two interproximal sites (not on the same tooth). Alternatively, it can be defined as a PPD of 5 mm or more in at least one interproximal site. The TLP-AA kit score for mild periodontal disease is 1.0 (1.0–1.5), which makes it indistinguishable from “no periodontal disease” [5], suggesting absence of symptoms and, thus, that, even in cases of mild periodontal disease, detection may fail owing to the limited sensitivity of the TLP-AA kit or insufficient enzyme production by the bacteria.

The Japanese Ministry of Health, Labour, and Welfare (MHLW) has surveyed the percentage of people with periodontal pockets measuring 4 mm or more. This criterion considers cases in which the pocket depth is 4 mm or greater at one or more sites. As this definition is broader than the CDC/AAP definition of mild periodontitis, it would yield an estimated prevalence of periodontal disease higher than under the CDC/AAP definition. Among Japanese individuals aged 15–24 (the same age group as the participants in this study), 17.8% (95% CI: 10.9–26.7%) reported having periodontal pockets measuring 4 mm or more [11]. In this study, participants with a TLP-AA kit score of 1.5 or higher were classified as positive, whereas red complex species were classified as positive. The proportion of positive participants was greater than 17.8%, though not statistically significantly. Since the MHLW survey examined PPD and this study examined the presence of red complex species, the two cannot be compared directly. However, as the MHLW survey estimates periodontal disease prevalence more broadly than the CDC/AAP definition, the TLP-AA kit score correlates with periodontal disease severity according to the CDC/AAP definition; Japanese athletes may have a higher prevalence of red complex species or periodontal disease than do Japanese non-athletes of the same age group.

A study of elite UK athletes playing a variety of sports found that erosive tooth wear was significantly more prevalent among male athletes (48.7%; n = 236) than female ones (28.4%; n = 116). Additionally, more males than females reported having good self-assessed oral health [3]. Furthermore, the odds ratio for caries was significantly higher for rugby (61.1%; n = 72) than for rowing (33.3%; n = 60) [3]. In contrast, this study observed no significant differences in periodontal risk by gender or sport, although the positive rate was higher in volleyball than in basketball and athletics. Since the participants had similar ages and environments, the differences between genders and sports may have been minimized. Future studies involving several facilities and athletes of differing ages could clarify the differences in periodontal risk among Japanese athletes by gender and sport.

The study cited above on elite British athletes assessed the impact on QOL using three items highly relevant to young, healthy populations: difficulty eating or drinking, relaxation including sleep, and smiling or laughing without embarrassment [3]. These items were adopted from the OIDP assessment scale used in the Adult Dental Health Survey for England, Wales, and Northern Ireland [9]. The study also assessed the impact on sport performance using the Overuse Injury Questionnaire [4] from OSTRC [3], which was developed for sports injury epidemiology research [4]. It has been confirmed to be highly sensitive and effective in recording patterns of acute injuries, overuse injuries, and illnesses across diverse athlete populations through a 40-week study of Norwegian athletes preparing for the 2012 Olympic and Paralympic Games [12]. In addition, a systematic review of athletes’ self-reported impacts of injury and illness on performance confirmed the validity and utility of the OSTRC overuse injury questionnaire [13]. Therefore, this study also adopted these questionnaires.

Gallagher et al. compared the periodontal disease status scored at Grades 3 and 4 (periodontal pockets ≥ 3.5 mm) versus those with milder conditions (Grades 0–2), as defined by the British Academy of Periodontology and Implant Dentistry’s Basic Periodontal Examination (2016 edition) [3]. Overall, no significant associations were observed across any of the three OIDP or four OSTRC items, regardless of the gender or sport [3]. However, a meta-analysis of papers published up to 2022, including this study, found a significant association in 396 subjects from Gallagher et al. [3] and two studies by Needleman et al. [1,2]: athletes with periodontal disease had 1.55 times higher odds of experiencing “self-reported reduced sports performance” than healthy athletes (moderate quality of evidence) [14]. However, the participants of the above studies did not include Japanese individuals—a paper examining London Olympic participants included seven Asians, but it is unclear whether any of them were Japanese [1]. In this study, one item of the OIDP (smiling or laughing without embarrassment) showed no significant relationship overall, but when stratified by club, practitioners of athletics with positive periodontal risk had notably more problems than did those without negative risk. Additionally, one of the four OSTRC overuse injury questionnaire items, sports-related pain, showed significantly more problems among those with positive periodontal risk than those without. Therefore, periodontal risk may affect the self-reported QOL and sports performance of Japanese university athletes, much as has been reported for international elite athletes.

This study also used the ISQ, which is a simple questionnaire developed to evaluate the self-perceived immune status [10]. It obtains 5-point responses regarding experiences of seven illnesses; results are calculated as an ISQ score [10]. Moreover, responses regarding general health and immune functioning were collected with the 11-point NRS [10]. Research reports using the ISQ have been accumulating rapidly. A systematic review compiled in 2025 reviewed 38 independent studies reported from 14 countries [15]. Notably, consistent reports indicate that the ISQ score correlates with anxiety across a variety of populations [16,17,18,19,20]. However, none of these studies target athletes or examine the association with periodontal disease. Therefore, we used the ISQ based on the hypothesis that red complex bacteria detected by the TLP-AA kit induce inflammation and affect the immune status. This study found no association between ISQ scores and periodontal risk. However, while no significant overall relationship was observed between general health and immune functioning as assessed by NRS, stratification by club revealed that athletes from the athletics club with periodontal risk reported significantly fewer positive responses than those without risk, suggesting that periodontal risk may affect immune status.

This study is significant because it is the first to investigate periodontal risk in Japanese athletes using the non-invasive TLP-AA kit. Additionally, it builds upon previous studies by evaluating the risk based on the presence of red complex species, a biological indicator that cannot be detected by traditional probing examinations.

However, this study has several limitations. First, the validity of the TLP-AA kit for young, healthy athletic populations has not yet been examined, and the highest TLP-AA kit score observed in this study was 2, which only 5% of the subjects obtained; none scored 3 or higher. Consequently, scores of 1.5–2 were combined and classified as indicating positive periodontal risk. Because the number of participants with severe periodontal disease was low, clear associations might not be possible with the OIDP, OSTRC Overuse Injury Questionnaire, or ISQ. In addition, OIDP, OSTRC Overuse Injury Questionnaire, and ISQ have not been examined, which limits comparisons with earlier studies. Additionally, since these questionnaires all rely on self-reporting, they may be subject to self-reporting bias. Moreover, exploratory analyses were performed in this study, stratified by gender and club. However, all were single-hypothesis tests without multiple comparison corrections (e.g., Bonferroni). Therefore, type I errors may have occurred. Furthermore, earlier studies reporting a high periodontal disease prevalence among athletes [1,2,3] focused on elite athletes such as Olympic competitors, whereas this study targeted athletes at a single university. Differences in competitive levels and the environment of the university also affected the prevalence of periodontal risk and its association with the questionnaire results. This exploratory study examined periodontal risk and its effects but did not investigate its causes or objective impact on quality of life. Therefore, future developments of this study are expected to reveal the environmental and psychological factors underlying periodontal risk in Japanese athletes. The goal is to develop treatment plans and recommendations that prevent or manage the condition and enhance athletic performance. To that end, extensive research involving a larger population of athletes is necessary.

## 5. Conclusions

This study exhibited a numerically higher prevalence of periodontal risk (TLP-AA kit score ≥ 1.5) in Japanese university athletes than the periodontal disease prevalence (defined by the Ministry of Health, Labour and Welfare’s 4 mm pocket criterion) of the same age group. Additionally, some analyses have suggested that periodontal risk may negatively impact self-reported QOL, sports performance, and immune status. However, studies involving larger athlete populations are needed to confirm these results.

## Figures and Tables

**Table 1:** The characteristics of the participants.

		Female				Male			
	Club	n	Mean	SD	95% CI	n	Mean	SD	95% CI
Age (yr)	Basketball	36	19.5	1.1	19.1–19.9	59	19.6	0.9	19.4–19.8
	Volleyball	38	20.1	1.2	19.7–20.5	37	20.3	1.4	19.9–20.8
	Athletics	59	20.0	1.1	19.7–20.3	84	20.2	1.1	20.0–20.5
	Total	133	19.9	1.2	19.7–20.1	180	20.0	1.2	19.9–20.2
Height (cm)	Basketball	36	164.7	5.0	163.0–166.4	59	177.3	6.2	175.7–178.9
	Volleyball	38	167.1	7.3	164.7–169.5	37	179.5	7.5	177.0–182.0
	Athletics	59	161.6	5.5	160.1–163.0	84	174.4	6.2	173.1–175.8
	Total	133	164.0	6.4	162.9–165.1	180	176.4	6.7	175.4–177.4
Weight (kg)	Basketball	36	60.6	6.4	58.4–62.7	59	71.3	6.6	69.6–73.1
	Volleyball	38	60.7	7.2	58.4–63.1	37	70.9	8.0	68.2–73.6
	Athletics	59	54.8	8.3	52.6–56.9	84	63.3	9.3	61.2–65.3
	Total	133	58.0	8.0	56.7–59.4	180	67.5	9.1	66.1–68.8

**Table 2:** Distribution of trypsin-like peptidase activity assay kit (TLP-AA kit) scores stratified by gender and club in university athletes (*n* = 313).

		TLP-AA Kit Score				
Gender	Club	1	1.5	1.75	2	Total
Female	Basketball	29	6	0	1	36
	Volleyball	22	8	6	2	38
	Athletics	44	8	3	4	59
	Sub-total	95	22	9	7	133
	%	71.4%	16.5%	6.8%	5.3%	100.0%
Male	Basketball	43	8	3	5	59
	Volleyball	28	5	2	2	37
	Athletics	66	8	8	2	84
	Sub-total	137	21	13	9	180
	%	76.1%	11.7%	7.2%	5.0%	100.0%
	Total	232	43	22	16	313
	%	74.1%	13.7%	7.0%	5.1%	100.0%

**Table 3:** Association between periodontal risk status (Positive/Negative) and Self-Reported Oral Impacts on Daily Performance (OIDP) scores.

		Periodontal Risk		
Question	Answer	Negative	Positive	*p*
Difficulty eating/drinking	No problem	206 (88.8%)	69 (85.2%)	0.430
	Difficulty	26 (11.2%)	12 (14.8%)	
Relaxing including sleeping	No problem	171 (73.7%)	62 (76.5%)	0.660
	Difficulty	61 (26.3%)	19 (23.5%)	
Smiling, laughing, or showing teeth without embarrassment	No problem	172 (74.1%)	53 (65.4%)	0.152
	Difficulty	60 (25.9%)	28 (34.6%)	

**Table 4:** Association between periodontal risk status and dichotomized self-rated general health and immune functioning scores (NRS).

		Periodontal Risk		
Question	Answer	Negative	Positive	*p*
Have you had any problems participating during past week?	Unaffected	227 (97.8%)	80 (98.8%)	1.000
	Affected	5 (2.2%)	1 (1.2%)	
To what extent have you reduced your training volume over the past week?	Unaffected	204 (87.9%)	69 (85.2%)	0.563
	Affected	28 (12.1%)	12 (14.8%)	
To what extent have problems reduced your performance during the past week?	Unaffected	219 (94.4%)	76 (93.8%)	0.788
	Affected	13 (5.6%)	5 (6.2%)	
To what extent have you experienced pain related to your sport during the past week?	Unaffected	212 (91.4%)	66 (81.5%)	0.016 *
	Affected	20 (8.6%)	15 (18.5%)	

Values are presented as the number of participants (*n*) and percentage (%) within each periodontal risk category. The self-rated scores (NRS) were dichotomized into the categories “Poor” (NRS < 7) and “Good” (NRS ≥ 7). The association between periodontal risk status (Positive/Negative) and each dichotomized NRS score was assessed using Fisher’s Exact Test. * An asterisk indicates a statistically significant difference in proportions between the positive and negative periodontal risk groups (*p* < 0.05).

## Data Availability

The data presented in this study are available upon request from the corresponding author.

## Abbreviations

The following abbreviations are used in this manuscript:

TLP-AA kit	Trypsin-Like Peptidase activity assay kit
OIDP	Oral Impacts on Daily Performance
OSTRC	Oslo Sports Trauma Research Centre
ISQ	Immune Status Questionnaire
NRS	Numerical Rating Scale
PPD	Periodontal Pocket Depth
CI	Confidence Interval
